# National Medical Convention in Philadelphia

**Published:** 1840

**Authors:** 


### NATIONAL MEDICAL CONVENTION.

At the second general Convention of the physicians of the State of Ohio, assembled at Columbus, on the first Monday of January, 1838, the following resolutions were unanimously adopted:

1. *Resolved*, That in the opinion of this Convention, the sessions of the different Medical Schools, throughout the Union, are too short, and that they ought to be extended one month, and the students required to stay to the *end* of the term.

2. *Resolved*, That the number of Professorships is too few, and that ampler provision should be made for teaching Physiology, Pathological Anatomy, Pharmacy, and the Natural History of Medicines, Botany, comparative Anatomy, Meteorology, Medical Jurisprudence, and Mental Physiology.

3. *Resolved*, That, if practicable, our Medical Schools should be so organized, as that Students, in their first course, would have their attention chiefly directed upon special Anatomy, Physiology, Chemistry, Pharmacy, and the other elementary branches; and their second upon Pathological Anatomy, Therapeutics, the practice of Physic, Surgery and Obstetrics.

4. *Resolved*, That in admitting Candidates to examination for degrees, a stricter regard than is at present shown, should be had to their preliminary education.

5. *Resolved*, That the practice of graduating young men before they are 21 years of age should be abandoned.

6. *Resolved*, That no Pupil ought to be graduated before the end of four years, from the time he commenced the study of Medicine.

7. *Resolved*, That, if the various Schools of the Union, were to send representatives to a meeting at some central point, to confer together, many of their existing defects, by a simultaneous, co-operative effort, might be successfully remedied, and that we, respectfully, recommend such a Convention to be held. Till when it would not be practicable, nor should it be expected that any single institution will attempt the reforms which are here proposed.

8. *Resolved*, That the corresponding Secretary be instructed to send a printed copy of the proceedings of this Convention, to all the Medical Institutions of the United States, with a letter, calling the attention of their Professors to these Resolutions.

Subsequently, without seeing fit to refer to these proceedings, the Medical Society of the State of New York, proposed a convention to be holden in the city of Philadelphia, on the 12th of May, 1840. As far as we have been able to learn, but few of our schools have selected their representatives to this meeting. But what excuse can they make for the omission? Is it not undeniable, that great and radical defects exist (we speak not of professors) in all of our institutions? Defects and errors, which depress the profession, are easily removable by concerted efforts, though incurable, by the exertions of any single school? Is it not universally admitted, that our sessions are too short, and that in many of our Colleges the number of Chairs is too few? These evils are certainly remediable, and their correction would be instantly followed by an elevation of character, in the younger members of the profession.

In the Medical Library for May, Dr. Dunglison states that this convention turned out an abortion. He says "not even the mover of the resolution in the Medical Society of the State of New York was present, and but few delegates presented themselves, among whom was Dr. Beck, of Albany—the well known author of the best book we possess on Medical Jurisprudence." Thus an object of universal interest to the profession is for the present defeated. We trust, however, that it is but a temporary defeat, and that the advocates of a higher standard of medical education in the United States, will soon meet together and remedy the defects of which all complain. The time for reform has assuredly arrived—the cry for it comes up from all parts of the country. D.

